# Dynamic Functional Connectivity Within the Fronto-Limbic Network Induced by Intermittent Theta-Burst Stimulation: A Pilot Study

**DOI:** 10.3389/fnins.2019.00944

**Published:** 2019-09-13

**Authors:** Yingying Tang, Xiong Jiao, Junjie Wang, Tianyuan Zhu, Jie Zhou, Zhenying Qian, Tianhong Zhang, Huiru Cui, Hui Li, Xiaochen Tang, Lihua Xu, Ling Zhang, Yanyan Wei, Jianhua Sheng, Liu Liu, Jijun Wang

**Affiliations:** ^1^Shanghai Key Laboratory of Psychotic Disorders, Shanghai Mental Health Center, Shanghai Jiao Tong University School of Medicine, Shanghai, China; ^2^Key Laboratory of Embedded System and Service Computing, Ministry of Education, Tongji University, Shanghai, China; ^3^School of Biomedical Engineering, Shanghai Jiao Tong University, Shanghai, China; ^4^Institute of Mental Health, Suzhou Guangji Hospital, The Affiliated Guangji Hospital of Soochow University, Suzhou, China; ^5^Department of Nuclear Medicine, Shanghai Chest Hospital, Shanghai Jiao Tong University, Shanghai, China; ^6^CAS Center for Excellence in Brain Science and Intelligence Technology (CEBSIT), Chinese Academy of Sciences, Shanghai, China; ^7^Institute of Psychology and Behavioral Science, Shanghai Jiao Tong University, Shanghai, China

**Keywords:** transcranial magnetic stimulation, intermittent theta-burst stimulation, after-effects, functional connectivity, the fronto-limbic network

## Abstract

**Purpose:**

The utility of transcranial magnetic stimulation (TMS) has been growing rapidly in both neurocognitive studies and clinical applications in decades. However, it remains unclear how the responses of the stimulated site and the site-related functional network to the external TMS manipulation dynamically change over time.

**Methods:**

A multi-session combining TMS-fMRI experiment was conducted to explore the spatiotemporal effects of TMS within the fronto-limbic network. Ten healthy volunteers were modulated by intermittent theta-burst stimulation (iTBS) at a precise site within the left dorsolateral prefrontal cortex (DLPFC, MNI coordinate [-44 36 20]), navigated by individual structural MRI image. Three-session resting-state fMRI images were acquired before iTBS (TP1), immediately after iTBS (TP2), and 15 min after iTBS (TP3) for each participant. Seventy-four regions of interests (ROIs) within the fronto-limbic network were chosen including the bilateral superior frontal gyrus (SFG), middle frontal gyrus (MidFG), inferior frontal gyrus (IFG), orbital gyrus (OrG), cingulate gyrus (CG), and subcortical nuclei (hippocampus and amygdala). Regional fractional amplitude of low-frequency fluctuation (fALFF) and ROI-to-ROI functional connectivity (FC) were compared among TP1, TP2, and TP3.

**Results:**

The immediate iTBS effect was observed at the stimulated site. FC between the left dorsolateral SFG and left dorsal IFG and between the left rostral IFG and right MidFG increased at TP2 as compared to at TP1 (all FDR-*p* < 0.05), while FC within the left OrG decreased. The relatively long-term iTBS effect transmitted with decreased FC between the left IFG and right amygdala, increased FC between the left MidFG and left OrG, and decreased FC between bilateral IFG and OrG at TP3 than at TP1 (all FDR-*p* < 0.05). Meanwhile, mean fALFF values over the left SFG, MidFG, ventral CG, and IFG were significantly increased at TP3 as compared to those at TP2 (all *p* < 0.05 with Bonferroni correction).

**Conclusion:**

By combining TMS and fMRI, it becomes possible to track the spatiotemporal dynamics of TMS after-effects within the fronto-limbic network. Our findings suggested that the iTBS effect dynamically changed over time from the local neural activation at the stimulated site to its connected remote regions within the fronto-limbic network.

## Introduction

Transcranial magnetic stimulation (TMS) provides a non-invasive way to explore the brain function in both basic neuroscience and clinical applications (Lefaucheur et al., 2014). TMS pulses induce current within the cortex underneath the site of stimulation, leading to local neural activation (Allen et al., 2007) as well as consequent alterations within a distributed network (Chen et al., 2013). After one-session TMS modulation, i.e., intermittent theta burst stimulation (iTBS), the after-effects can be beyond the duration of stimulation and may last about 30 min (Huang et al., 2005). Therefore, the TMS after-effects should be temporally and spatially dynamic over the local region and within the whole-brain network (Ruff et al., 2009; Hawco et al., 2017, 2018). However, the pattern of tempo-spatial dynamics induced by TMS manipulation remains unclear.

There is few evidence of the duration of TMS after-effects over the primary motor cortex in humans. Huang et al. examined the after-effects induced by different TMS paradigms (iTBS, continuous TBS, intermediate TBS, and 15 Hz TMS) using motor evoked potential (MEP) amplitude (Huang et al., 2005). The enhanced MEP amplitudes last about 20 min after 600-pulse iTBS, but 60 min after 600-pulse cTBS (Huang et al., 2005). Peinemann et al. (2004) found one-session 5 Hz repetitive TMS with 1,800 pulses rather than 150 pulses induced stable MEP facilitatory for at least 30 min. To further clarify the TMS after-effects beyond the motor cortex, other neuroimaging techniques are required to measure the TMS induced neural activity.

Combining EEG with TMS is one way to characterize the temporal neural processing following TMS manipulation in non-motor regions (Chung et al., 2015). A review by Chung et al. proposed the TMS-evoked potential (TEP) as a sensitive measure of cortical excitation and inhibition (Chung et al., 2015). Indicators such as N100 and long-interval cortical inhibition (LICI) developed from TEP over the dorsolateral prefrontal cortex (DLPFC) were proven to be helpful for reflecting the integrity of the frontal cortex and had potentials for predicting the outcome of treatment in depression (Sun et al., 2016). When the time varied after a TMS pulse, distinctive TEP components occurred with dynamic topographic representation (Chung et al., 2015). However, it is lack of multi-session off-line EEG-TMS to monitor the affect-effects along with time following TMS. On the other hand, although topographic distribution for different TEP components suggested that neural activations not only at the site of stimulation but also at the distal sites could be influenced by TMS, more direct evidence of the precise spatial dynamics induced by TMS is further needed.

Some additional attempts have been made by combining resting-state fMRI (rsfMRI) with TMS to obtain a better spatial resolution. Wang et al. (2014) aimed to enhanced the hippocampus-related associative memory that was achieved by delivering multi-session 20 Hz rTMS over the lateral parietal cortex with strong functional connectivity (FC) with hippocampus. Chen et al. directly examined the dynamic interaction within three large-scale neural networks, i.e., the default mode network (DMN), the salience network (SN), and the central executive network (ECN) (Chen et al., 2013). TMS targets in different network nodes had causal influence to both within-network and between-network connectivity (Chen et al., 2013). Hawco et al. suggested spread TMS-induced cortical changes that were related to the FC between the stimulated site and SN (Hawco et al., 2018). Moreover, combining TMS and fMRI has attracted increasing attention in optimizing the rTMS treatments (Fox et al., 2012a, b, 2014), with the most mature example of major depressive disorder (MDD). As the left DLPFC was one of the most popular targets for rTMS treatment in MDD, individuals’ intrinsic FC between this site and the subgenual anterior cingulate cortex (sgACC) predicted the efficacy of their rTMS treatments (Weigand et al., 2018). Therefore, it is worthy of uncovering the spatial propagation of TMS after-effects within the cortico-subcortical networks to improve the therapeutic outcome.

In the present study, we performed single-session rsfMRI acquisition before iTBS and two-session rsfMRI acquisitions after iTBS to characterize the immediate and long-term after-effects within and beyond the site of stimulation in healthy volunteers. Precise neuroimaging-guided iTBS was delivered over the left DLPFC (the most popular TMS target) using an MRI-compatible TMS coil within the MRI scanner, which made it possible to monitor the immediate iTBS effect. Considering the limitation of a small sample size, we restricted our analysis within the fronto-limbic network to reflect the spatial propagation following iTBS. Firstly, the fronto-limbic network is well known, consisting of a dorsal pathway with DLPFC and anterior cingulate cortex (ACC) for emotional regulation and executive control, as well as a ventral pathway with orbital frontal cortex, hippocampus, and amygdala for reward processing, both playing an important role in the etiology of MDD (Mayberg, 2003). Secondly, previous studies on TMS over the left DLPFC suggested a prominent effect within the fronto-limbic network, especially the FC between the left DLPFC and ACC, in both healthy controls and patients with depression (Fox et al., 2012a; Tik et al., 2017). We hypothesized that (1) the immediate iTBS effect may be stronger around the site of stimulation than in remote sites, and (2) the long-term iTBS effect may spread beyond the site of stimulation and be stronger in the other node(s) within the fronto-limbic network.

## Materials and Methods

### Participants

**FIGURE 1:**
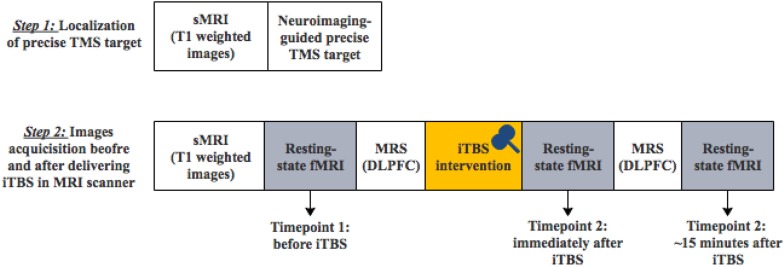
The diagram of the two-step experimental procedure (sMRI, structural MRI; TMS, transcranial magnetic stimulation; MRS, magnetic resonance spectroscopy).

The experimental protocol was approved by the Ethics Committee at Shanghai Mental Health Center. Written informed consent was obtained from each participant. Ten healthy volunteers [4 females and 6 males; age (mean ± SD): 25.5 ± 2.8 years old, education (mean ± SD): 17.0 ± 0.8 years] were recruited from the community by online advertisement. All participants were screened by a psychiatrist. Exclusion criteria include a personal or family history of mental illness, a history of any substance or alcohol abuse, severe physical disease, loss of consciousness, any foreign metallic objects in their head or stimulator in their body, or any other contraindication for MRI examination or TMS intervention. All participants received one-session TMS modulation and completed MRI image acquisitions (Figure 1).

### TMS Procedure

Transcranial magnetic stimulation stimuli were delivered using MagPro X100 magnetic stimulator (Medtronic Co., Denmark). Individual resting motor threshold (MT) was examined with a figure-eight coil (MC-B70) outside the MRI scanner. MEP was measured from the left abductor pillicis brevis (APB) muscle with surface electrodes and then amplified and quantified using Keypoint (Medtronic Co., Denmark). The “hotspot” for each participant was determined over the primary motor area where the largest MEP was evoked (Tang et al., 2018). In 5 out of 10 trials, the lowest intensity that succeeded in evoking peak-to-peak MEP exceeding 50 μV was defined as individual MT.

One-session iTBS was applied in the MRI scanner using an MRI-compatible coil (MRi-B91). The iTBS applied three 50-Hz pulses every 200 ms as one burst and delivered for 2 s with 10-s intervals (Huang et al., 2005). In total, there were 600 pulses for each session. The intensity of iTBS was set as 80% of individual resting MT (Grossheinrich et al., 2009; Rossi et al., 2009; Lefaucheur et al., 2014). The target was precisely localized over individual left DLPFC determined by the MNI coordinate (*x* = −44, *y* = 36, *z* = 20) using LOCALITE TMS Navigator (LOCALITE GmbH, Schloss Birlinghoven, Germany) (Lerman et al., 2014). The localization of left DLPFC was at the border of BA 9 and 46 defined by Rajkowska and Goldman-Rakic (1995). The TMS coil was fixed inside the MRI head coil.

### MRI Data Acquisition

All MRI images were obtained in Shanghai Mental Health Center on a Siemens 3T Verio MRI system (MR B17, Siemens AG, Erlangen, Germany) with an open 1-channel head coil. Each subject completed two-step MRI scans depicted in Figure 1: (1) the first MRI scan only included structural T1-weighted images for the localization of individual precise TMS target (the left DLPFC); 2) after the fixation of TMS coil, the second MRI scan included structural T1-weighted images, the first resting-state fMRI images before iTBS modulation, magnetic resonance spectroscopy (MRS), the second resting-state fMRI images immediately after iTBS modulation, MRS and the third resting-state fMRI images 15 min after iTBS modulation. MRS data analysis was not included in the current study.

T1-weighted images were acquired using a magnetization-prepared rapid acquisition gradient-echo (MPRAGE) sequence with repetition time (TR) = 2530 ms, echo time (TE) = 3.65 ms, field of view (FOV) = 256 mm, matrix = 256 × 256, slice thickness = 1 mm, 224 coronal slices, flip angle = 7°, and generalized autocalibrating partial parallel acquisition (GRAPPA) with acceleration factor 2.

Resting-stage fMRI images were acquired using an echo planar scanning sequence with TR = 2,000 ms, TE = 30 ms, FOV = 220 mm, matrix = 64 × 64, slice thickness = 4 mm, 30 axial slices with a between-slice gap of 1 mm, flip angle = 90°, and total data volume = 180. Subjects were asked to close their eyes, relax, and think of nothing during the rsfMRI acquisitions.

### Resting-State fMRI Data Processing

Resting-state fMRI data were preprocessed including slice timing correction, realignment, normalization, and smoothing (8-mm FWHM Gaussian filter) using CONN v.18.b^1^ (Whitfield-Gabrieli and Nieto-Castanon, 2012). All subjects’ functional data were co-registered to their structural data with a linear transformation and then normalized to MNI space with a non-linear transformation. The Artifact Detection Tools (ART) were embedded in CONN to identify outlier images if the head motion in *x*, *y*, or *z* direction was over 1 mm or the global mean intensity in the image was over three standard deviations from the mean image intensity for all images. Individual T1-weighted images were segmented into gray matter, white matter, and CSF and generated three masks (Whitfield-Gabrieli et al., 2016). Linear regression was applied to remove the confounding effects including (1) BOLD signals from white matter and CSF, which were used for aCompCor; (2) head motion confound defined by six rigid-body motion parameters and six first-order temporal derivatives; (3) ART-based scrubbing parameters containing invalid scans. Then a band-pass filter (0.01–0.08 Hz) and linear detrending were applied to the resulting residual BOLD time series.

Firstly, ROI-to-ROI FC analysis was performed at individual level using Conn. Seventy-four frontal, limbic, and subcortical ROIs were selected from the Human Brainnetome Atlas (Fan et al., 2016), including 14 ROIs in superior frontal gyrus (SFG), 14 ROIs in middle frontal gyrus (MidFG), 12 ROIs in inferior frontal gyrus (IFG), 12 ROIs in orbital gyrus (OrG), 14 ROIs in limbic lobe/cingulate gyrus (CG), and 8 subcortical nuclei (4 in amygdala and 4 in hippocampus) with detailed information in Supplementary Material.

Secondly, amplitude of low-frequency fluctuation (ALFF) and fractional ALFF (fALFF) were calculated within each voxel using Conn (Zang et al., 2007; Zou et al., 2008). The fALFF at each voxel was the relative amplitude of BOLD signal fluctuation in the frequency band of 0.01–0.08 Hz compared to the entire frequency band before filtering (Whitfield-Gabrieli and Nieto-Castanon, 2012). Further, mean fALFF values were calculated within each of the above 74 ROIs.

### Statistical Analysis

There were three conditions for each participant including before the iTBS (TP1), immediately after the iTBS (TP2), and 15 min after the iTBS (TP3). For ROI-to-ROI FC values, the immediate iTBS effect was examined by comparing FC values at TP2 with those at TP1, while the long-term iTBS effect was examined by comparing FC values at TP3 with those at TP1. Two-sided paired-sample *t* tests were performed for both the ROI-to-ROI FC. The statistical significance was set at a false discover rate-corrected *p* (FDR-*p*) < 0.05.

Repeated-measure analyses of variance (ANOVAs) were performed for mean regional fALFF values with two within-group factors of time (TP1, TP2, and TP3) and region (74 ROIs). Simple-effect tests were further performed to examine regional fALFF differences across TP1, TP2, and TP3. Multiple comparisons were controlled using Bonferroni correction.

## Results

### The iTBS Effect on ROI-to-ROI FC

**FIGURE 2:**
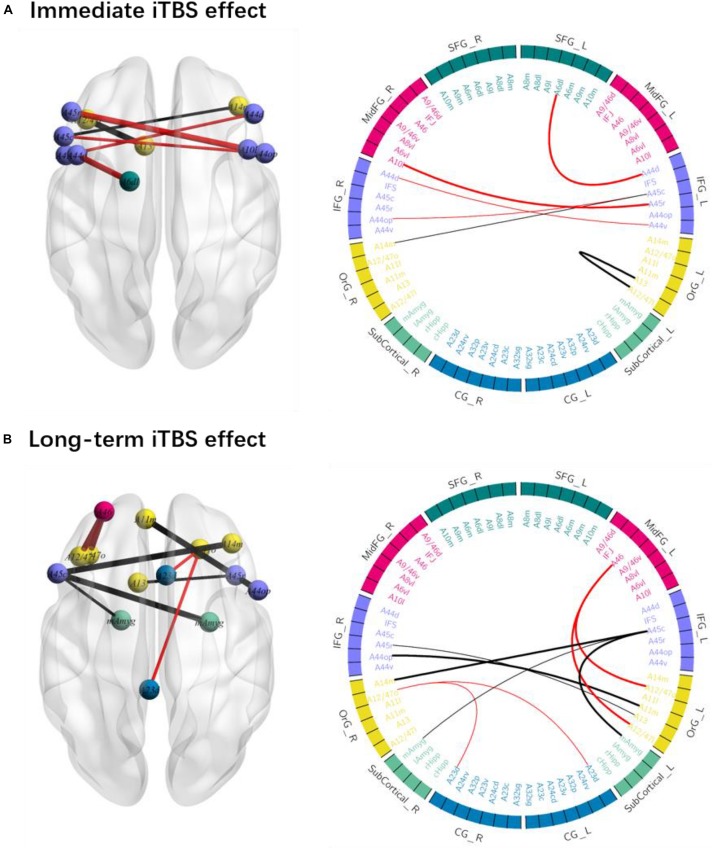
The immediate and long-term iTBS after-effects on the ROI-to-ROI functional connectivity (FC). **(A)** FC within the left frontal regions and between the bilateral areas changed at timepoint 2 as compared to that at timepoint 1. **(B)** FC within the bilateral frontal areas and between the left IFG and amygdala changed at timepoint 3 as compared to that at timepoint 1. FC increases are in red and FC decreases are in black (thick line: *p* < 0.05; thin line: *p* < 0.1).

The iTBS effect was dynamic along with the time and spread within the fronto-limbic network as shown in Figure 2 and Table 1.

**TABLE 1:** The ROI-to-ROI functional connectivity significantly changed after the intermittent theta-burst stimulation (iTBS) immediately and 15 min later.

**ROI-to-ROI functional connectivity**	**Statistic**	**Uncorrected *p* value**	**FDR-corrected *p* value**
***Immediate iTBS effect (TP2 vs. TP1):***			
**Left dorsolateral SFG–left dorsal IFG**	***F*(1,9)** = **48.92**	**0.0001**	**0.0046**
**Left OrG–left lateral OrG**	***F*(1,9) = 33.97**	**0.0003**	**0.0183**
**Left rostral IFG–right lateral MidFG**	***F*(1,9) = 28.98**	**0.0004**	**0.0323**
Left ventral IFG–right dorsal IFG	*F*(1,9) = 24.90	0.0007	0.0547
Left caudal IFG–right opercular IFG	*F*(1,9) = 19.89	0.0016	0.0576
Left caudal IFG–right medial OrG	*F*(1,9) = 20.82	0.0014	0.0993
***Long-term iTBS effect (TP3 vs. TP1):***			
**Left caudal IFG–right medial amygdala**	***F*(1,9)** = **29.45**	**0.0004**	**0.0153**
**Left caudal IFG–right medial OrG**	***F*(1,9)** = **31.58**	**0.0003**	**0.0153**
**Left medial OrG–right opercular IFG**	***F*(1,9)** = **26.44**	**0.0006**	**0.0445**
**Left MidFG–left lateral OrG**	***F*(1,9)** = **26.37**	**0.0006**	**0.0449**
**Left MidFG–left orbital OrG**	***F*(1,9)** = **26.98**	**0.0006**	**0.0415**
Right orbital OrG–left dorsal CG	*F*(1,9) = 24.58	0.0008	0.0571
Left caudal IFG–left medial amygdala	*F*(1,9) = 14.87	0.0039	0.0941
Left OrG–right rostral IFG	*F*(1,9) = 20.92	0.0013	0.0977
Right orbital OrG–right CG	*F*(1,9) = 16.78	0.0027	0.0982

*CG, cingulate gyrus; FDR, false discovery rate; FG, inferior frontal gyrus; iTBS, intermittent theta-burst stimulation; MidFG, middle frontal gyrus; OrG, orbital gyrus; ROI, region of interest; SFG, superior frontal gyrus; TP, timepoint. Significant FC changes with FDR-*p* < 0.05 are in bold.*

The immediate iTBS effect was observed within the left frontal areas and between the bilateral frontal areas. FC between the left dorsolateral SFG (A6dl_l) and left dorsal IFG (A44d, 0.110 ± 0.055, FDR-*p* = 0.005) and between the left rostral IFG (A45r_l) and right MidFG (A10l_r, 0.353 ± 0.032, FDR-*p* = 0.032) increased at TP2 as compared to those at TP1 (−0.038 ± 0.064 and 0.170 ± 0.043). FC between the left OrG areas (A13_l and A12_47l_l, −0.047 ± 0.059, FDR-*p* = 0.018) decreased at TP2 as compared to that at TP1 (0.178 ± 0.043). The increased FC between the left dorsal SFG and left dorsal IFG and decreased FC between the left OrG areas were presented in each of the 10 participants, suggesting a high consistency at the individual level (Figure 2C). However, the above iTBS effect was attenuated not significant around 15 min after the iTBS modulation.

The long-term iTBS effect on the FC was more widespread within the bilateral frontal areas and between left IFG and amygdala as shown in Figure 2B. FC between the left caudal IFG (A45c_l) and right medial amygdala (mAmyg_r) significantly decreased around 15 min after the iTBS (−0.215 ± 0.057, FDR-*p* = 0.015) as compared to that before the iTBS (0.035 ± 0.063), but not significantly changed immediately after the iTBS (FDR-*p* > 0.05). FC between the left caudal IFG (A45c_l) and left medial amygdala (mAmyg_l) had a tendency to decrease in a similar way (FDR-*p* = 0.094). FC between the left MidFG (A46_l) and left OrG increased at TP3 (A12_47o_l: 0.152 ± 0.041, FDR-*p* = 0.042; A12_47l_l: 0.190 ± 0.063, FDR-*p* = 0.045) as compared to that at TP1 (A12_47o_l: −0.048 ± 0.048; A12_47l_l: 0.037 ± 0.053), whereas FC between the left caudal IFG (A45c_l) and right medial OrG (A14m_r, TP1: −0.059 ± 0.048; TP3: −0.271 ± 0.037; FDR-*p* = 0.015) and between the right opercular IFG (A44op_r) and left medial OrG (A11m_l, TP1: −0.127 ± 0.055; TP3: −0.382 ± 0.059; FDR-*p* = 0.045) decreased. Additionally, FC between the right OrG (A12_47o_r) and bilateral cingulate gyrus also had a tendency to be significantly changed around 15 min after iTBS (left CG: A23d_l, FDR-*p* = 0.057; right CG: A23d_r, FDR-*p* = 0.098).

### The iTBS Effect on Regional fALFF

**FIGURE 3:**
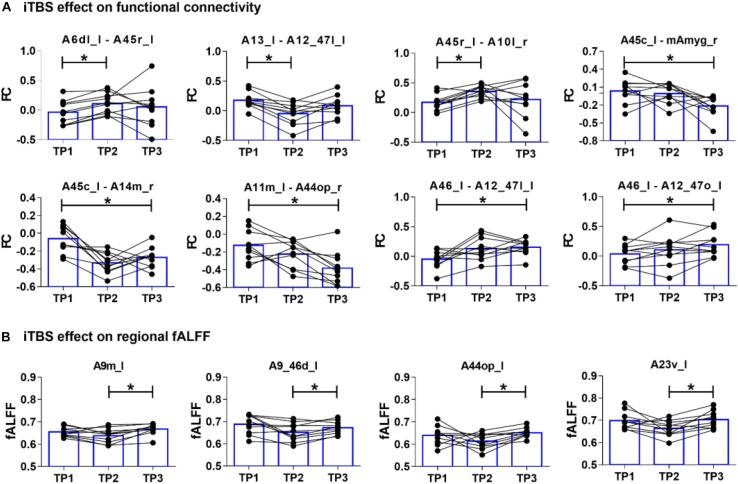
The iTBS-induced changes of functional connectivity and fractional amplitude of low-frequency fluctuation (fALFF) at timepoint 1 (TP1), timepoint 2 (TP2), and timepoint 3 (TP3). **(A)** Immediate iTBS effect (TP1 vs. TP2) was observed within the left frontal areas and between the bilateral frontal areas, while long-term iTBS effects (TP1 vs. TP3) was more widespread within the bilateral frontal areas and between the left caudal IFG (A45c_l) and right medial amygdala (mAmyg_r). **(B)** Regional fALFF changed from TP2 to TP3 at the left medial SFG (A9m_l), left dorsal MidFG (A9_46d_l), left opercular IFG (A44op_l), and left ventral cingulate gyrus (A23v_l).

There were significant main effects of both time [*F*(2,18) = 3.881, *p* = 0.047] and region [*F*(73,657) = 19.353, *p* < 0.001] on regional fALFF. As shown in Figure 3, simple effect tests further suggested that regional fALFF values over the left medial SFG (A9m_l, *p* = 0.010), left dorsal MidFG (A9_46d_l, *p* = 0.049), left ventral cingulate gyrus (A23v_l, *p* = 0.011), and left opercular IFG (A44op_l, *p* = 0.004) increased at TP3 as compared to those at TP2 (all *p* values <0.05 adjusted using Bonferroni correction). There were no significant differences of mean fALFF values between TP1 and TP2 or between TP1 and TP3.

## Discussion

By combining TMS with multi-session resting-state fMRI, the present study examined the dynamic changes within the fronto-limbic network induced by precise iTBS modulation. As we hypothesized, iTBS after-effects were observed not only around the stimulated region but also at remote regions interconnected with the target. Immediately after iTBS, FC changed significantly between the left frontal areas and between bilateral frontal areas. About 15 min later, the alteration of FC transmitted and was more widespread between bilateral frontal areas and between left IFG and amygdala. Further, fALFF measurements at two different time windows after iTBS showed increases at the left SFG, MidFG, CG, and IFG. More importantly, these spreading patterns after precise neuroimaging-guided iTBS manipulation had a high consistency at the individual level. Our findings suggested TMS-fMRI as an advanced technique to determine the dynamic patterns of TMS-induced effects within the brain network.

We delivered TMS pulses precisely at the dorsolateral prefrontal node (MNI coordinate, *x* = −44, *y* = 36, *z* = 20) in the left MidFG (A9_46v_l), which had structural connections with the right frontal regions and subcortical regions and FC with the fronto-parietal network according to the Human Brainnetome Atlas (Yeo et al., 2011; Fan et al., 2016). Immediately after single-session excitatory iTBS, increased FC between the left dorsal SFG and IFG and between left IFG and right MidFG may suggest an enhancement in the dorsal pathway, whereas decreased FC between the left OrG areas suggested an attenuation in the ventral pathway (Mayberg, 2003; Ruff et al., 2009; Eldaief et al., 2011). Consistent with previous findings, Hawco et al. found the prominent TMS-induced FC changes between the DLPFC target and salience network (SN) (Hawco et al., 2018). Chen et al. found excitatory single pulse over the anterior media frontal cortex increased within-SN FC (Chen et al., 2013). A previous TMS-PET study also showed significant dopaminergic changes in the medial orbitofrontal cortex and ACC after 10 Hz TMS over the left DLPFC in healthy volunteers (Cho and Strafella, 2009). An iTBS effect could spread out immediately from the target to the left SFG, IFG, and OrG.

The iTBS after-effect continued its propagation from the left frontal regions to bilateral amygdala and to right frontal regions along with time. These remote iTBS effects could be based on anatomical connectivity by white matter tracts, such as the uncinate fasciculus connecting the frontal region and the limbic system as well as the genu of corpus callosum connecting bilateral IFG (Kier et al., 2004; Fan et al., 2016). About 15 min later, decreased FC between the left IFG and bilateral amygdala suggested a subsequent change in the ventral pathway, which is critical for the generation and regulation of negative emotion (Hiser and Koenigs, 2018). More supports of remote effects induced by TMS were provided by the changed functional covariation between different regions. Wang et al. enhanced the cortico-hippocampal FC by multi-session excitatory TMS over the lateral parietal cortex (Wang et al., 2014). Other concurrent TMS-fMRI studies also found TMS-induced changes of remote FC, such as that between the frontal cortex/parietal and visual occipital regions (Ruff et al., 2006; Sack et al., 2007). Since the iTBS effect became more prominent within the left IFG later, we inferred that neural activation at the left IFG may be indirect as a consequence of limbic activation.

Regional fALFF values increased at the left SFG, MidFG, IFG, and ventral CG later after iTBS, which further supported the continuous excitatory effect at 15 min after iTBS. Few other studies examined the regional activity after TMS or iTBS modulation. Only Chen et al. reported decreased low-frequency signal amplitude within the DMN network after inhibitory rTMS (Chen et al., 2013). The finding of increased fALFF value in the left MidFG, SFG, and IFG was consistent with the more widespread FC connectivity induced by long-term iTBS effect. Additionally, the regional fALFF increased in the left ventral CG, and FC between the right OrG and bilateral CG also had a tendency to be significantly changed around 15 min after iTBS. Cingulate cortex was a hot hub for the connectivity within the DLPFC and limbic network (Tik et al., 2017). Fox et al. suggested that FC between the DLPFC and subgenual ACC could be a valuable predictor for the TMS effects in depression (Fox et al., 2012a). Tik et al. found only the connectivity to the ACC increased at 15 min after 10-Hz TMS modulation over the left DLPFC, and this effect disappeared at 30 min after TMS (Tik et al., 2017).

As shown in Figure 3, FC and fALFF changes from TP1 to TP2 varied among subjects, but the changes from TP2 to TP3 had a high inter-subject consistency. One critical factor contributing to the heterogeneity of TMS after-effects could be the different location of individual TMS targets (Fox et al., 2012a; Lefaucheur et al., 2014; Hawco et al., 2018). The use of individual T1-image-guided localization for the TMS target in the present study could reduce the heterogeneity of the iTBS effect. The other factor may be the different latency of individual TMS-induced response. A higher consistency for FC and fALFF changes among subjects at TP3 suggested a convergence of the remote iTBS effect within the fronto-limbic network after a longer time. However, more evidence is required in the future to prove our intention.

There were several limitations in the present study. Firstly, only 10 healthy volunteers were recruited for the experiments. The small sample size made it a bit difficult to obtain strong statistical significance. Secondly, there was no control or sham condition. As an exploratory study, only iTBS over the left DLPFC was used. The observed iTBS-induced dynamic patterns needed to be interpreted in caution. Thirdly, we narrowed our observation in the fronto-limbic network to tract the iTBS effects. There could be other regions or networks also influenced by iTBS that were omitted here. Further, more works with experimental designs including control/sham TMS condition, different intensities, and stimulated sites are needed to predict the iTBS-induced effects within the large-scale brain networks.

In conclusion, to the best of our knowledge, there were a few studies characterizing the temporal and spatial dynamics induced by single-session iTBS manipulation. The FC and fALFF measurements consistently demonstrated a significant immediate iTBS effect around the stimulated DLPFC site, while a long-term iTBS effect was prominent between the left IFG and amygdala as well as between bilateral frontal regions. Thus, our findings suggested that the combination of TMS-fMRI should be an advanced technique to clarify the TMS-induced effect, as well as to optimize the clinical application for treatments.

## Data Availability

The datasets generated for this study are available on request to the corresponding author.

## Ethics Statement

The experimental protocol was approved by the Ethics Committee at Shanghai Mental Health Center. Written informed consent was obtained from each participant.

## Author Contributions

JiW and YT conceptualized and designed the study. TZha, HC, HL, LX, and YW recruited the participants and completed the screening assessments. YT, JuW, TZhu, and ZQ performed the TMS manipulation. YT, XJ, XT, JS, and JiW analyzed the data and performed the statistical analysis. YT, JS, and JiW wrote the first draft of the manuscript. All authors revised the manuscript and approved the final manuscript.

## Conflict of Interest Statement

The authors declare that the research was conducted in the absence of any commercial or financial relationships that could be construed as a potential conflict of interest.

## Footnotes

1 https://www.nitrc.org/projects/conn

## References

[B1] Allen E. A. Pasley B. N. Duong T. Freeman R. D. (2007). Transcranial magnetic stimulation elicits coupled neural and hemodynamic consequences. *Science* 317 1918–1921. 10.1126/science.1146426 17901333

[B2] Chen A. C. Oathes D. J. Chang C. Bradley T. Zhou Z. W. Williams L. M. (2013). Causal interactions between fronto-parietal central executive and default-mode networks in humans. *Proc. Natl. Acad. Sci. U.S.A.* 110 19944–19949. 10.1073/pnas.1311772110 24248372 PMC3856839

[B3] Cho S. S. Strafella A. P. (2009). rTMS of the left dorsolateral prefrontal cortex modulates dopamine release in the ipsilateral anterior cingulate cortex and orbitofrontal cortex. *PLoS One* 4:e6725. 10.1371/journal.pone.0006725 19696930 PMC2725302

[B4] Chung S. W. Rogasch N. C. Hoy K. E. Fitzgerald P. B. (2015). Measuring brain stimulation induced changes in cortical properties using TMS-EEG. *Brain Stimul.* 8 1010–1020. 10.1016/j.brs.2015.07.029 26275346

[B5] Eldaief M. C. Halko M. A. Buckner R. L. Pascual-Leone A. (2011). Transcranial magnetic stimulation modulates the brain’s intrinsic activity in a frequency-dependent manner. *Proc. Natl. Acad. Sci. U.S.A.* 108 21229–21234. 10.1073/pnas.1113103109 22160708 PMC3248528

[B6] Fan L. Li H. Zhuo J. Zhang Y. Wang J. Chen L. (2016). The human brainnetome atlas: a new brain atlas based on connectional architecture. *Cereb. Cortex* 26 3508–3526. 10.1093/cercor/bhw157 27230218 PMC4961028

[B7] Fox M. D. Buckner R. L. Liu H. Chakravarty M. M. Lozano A. M. Pascual-Leone A. (2014). Resting-state networks link invasive and noninvasive brain stimulation across diverse psychiatric and neurological diseases. *Proc. Natl. Acad. Sci. U.S.A.* 111 E4367–E4375. 10.1073/pnas.1405003111 25267639 PMC4205651

[B8] Fox M. D. Buckner R. L. White M. P. Greicius M. D. Pascual-Leone A. (2012a). Efficacy of transcranial magnetic stimulation targets for depression is related to intrinsic functional connectivity with the subgenual cingulate. *Biol. Psychiatry* 72 595–603. 10.1016/j.biopsych.2012.04.028 22658708 PMC4120275

[B9] Fox M. D. Halko M. A. Eldaief M. C. Pascual-Leone A. (2012b). Measuring and manipulating brain connectivity with resting state functional connectivity magnetic resonance imaging (fcMRI) and transcranial magnetic stimulation (TMS). *Neuroimage* 62 2232–2243. 10.1016/j.neuroimage.2012.03.035 22465297 PMC3518426

[B10] Grossheinrich N. Rau A. Pogarell O. Hennig-Fast K. Reinl M. Karch S. (2009). Theta burst stimulation of the prefrontal cortex: safety and impact on cognition, mood, and resting electroencephalogram. *Biol. Psychiatry* 65 778–784. 10.1016/j.biopsych.2008.10.029 19070834

[B11] Hawco C. Armony J. L. Daskalakis Z. J. Berlim M. T. Chakravarty M. M. Pike G. B. (2017). Differing time of onset of concurrent TMS-fMRI during associative memory encoding: a measure of dynamic connectivity. *Front. Hum. Neurosci.* 11:404. 10.3389/fnhum.2017.00404 28855865 PMC5557775

[B12] Hawco C. Voineskos A. N. Steeves J. K. E. Dickie E. W. Viviano J. D. Downar J. (2018). Spread of activity following TMS is related to intrinsic resting connectivity to the salience network: a concurrent TMS-fMRI study. *Cortex* 108 160–172. 10.1016/j.cortex.2018.07.010 30195825

[B13] Hiser J. Koenigs M. (2018). The multifaceted role of the ventromedial prefrontal cortex in emotion, decision making, social cognition, and psychopathology. *Biol. Psychiatry* 83 638–647. 10.1016/j.biopsych.2017.10.030 29275839 PMC5862740

[B14] Huang Y. Z. Edwards M. J. Rounis E. Bhatia K. P. Rothwell J. C. (2005). Theta burst stimulation of the human motor cortex. *Neuron* 45 201–206. 10.1016/j.neuron.2004.12.033 15664172

[B15] Kier E. L. Staib L. H. Davis L. M. Bronen R. A. (2004). MR imaging of the temporal stem: anatomic dissection tractography of the uncinate fasciculus, inferior occipitofrontal fasciculus, and Meyer’s loop of the optic radiation. *AJNR Am. J. Neuroradiol.* 25 677–691. 15140705 PMC7974480

[B16] Lefaucheur J. P. Andre-Obadia N. Antal A. Ayache S. S. Baeken C. Benninger D. H. (2014). Evidence-based guidelines on the therapeutic use of repetitive transcranial magnetic stimulation (rTMS). *Clin. Neurophysiol.* 125 2150–2206. 10.1016/j.clinph.2014.05.021 25034472

[B17] Lerman C. Gu H. Loughead J. Ruparel K. Yang Y. Stein E. A. (2014). Large-scale brain network coupling predicts acute nicotine abstinence effects on craving and cognitive function. *JAMA Psychiatry* 71 523–530. 10.1001/jamapsychiatry.2013.4091 24622915 PMC4097018

[B18] Mayberg H. S. (2003). Modulating dysfunctional limbic-cortical circuits in depression: towards development of brain-based algorithms for diagnosis and optimised treatment. *Br. Med. Bull.* 65 193–207. 10.1093/bmb/65.1.193 12697626

[B19] Peinemann A. Reimer B. Loer C. Quartarone A. Munchau A. Conrad B. (2004). Long-lasting increase in corticospinal excitability after 1800 pulses of subthreshold 5 Hz repetitive TMS to the primary motor cortex. *Clin. Neurophysiol.* 115 1519–1526. 10.1016/j.clinph.2004.02.005 15203053

[B20] Rajkowska G. Goldman-Rakic P. S. (1995). Cytoarchitectonic definition of prefrontal areas in the normal human cortex: II. variability in locations of areas 9 and 46 and relationship to the talairach coordinate system. *Cereb. Cortex* 5 323–337. 10.1093/cercor/5.4.323 7580125

[B21] Rossi S. Hallett M. Rossini P. M. Pascual-Leone A. (2009). Safety, ethical considerations, and application guidelines for the use of transcranial magnetic stimulation in clinical practice and research. *Clin. Neurophysiol.* 120 2008–2039. 10.1016/j.clinph.2009.08.016 19833552 PMC3260536

[B22] Ruff C. C. Blankenburg F. Bjoertomt O. Bestmann S. Freeman E. Haynes J. D. (2006). Concurrent TMS-fMRI and psychophysics reveal frontal influences on human retinotopic visual cortex. *Curr. Biol.* 16 1479–1488. 10.1016/j.cub.2006.06.057 16890523

[B23] Ruff C. C. Driver J. Bestmann S. (2009). Combining TMS and fMRI: From ‘virtual lesions’ to functional-network accounts of cognition. *Cortex* 45 1043–1049. 10.1016/j.cortex.2008.10.012 19166996 PMC2726131

[B24] Sack A. T. Kohler A. Bestmann S. Linden D. E. Dechent P. Goebel R. (2007). Imaging the brain activity changes underlying impaired visuospatial judgments: simultaneous FMRI, TMS, and behavioral studies. *Cereb. Cortex* 17 2841–2852. 10.1093/cercor/bhm013 17337745

[B25] Sun Y. Farzan F. Mulsant B. H. Rajji T. K. Fitzgerald P. B. Barr M. S. (2016). Indicators for remission of suicidal ideation following magnetic seizure therapy in patients with treatment-resistant depression. *JAMA Psychiatry* 73 337–345. 10.1001/jamapsychiatry.2015.3097 26981889

[B26] Tang Y. Ying C. Wang J. Jiao X. Qian Z. Zhang T. (2018). Precise theta burst transcranial magnetic stimulation selectively reduced duration-related mismatch negativity. *Biol. Psychol.* 137 125–132. 10.1016/j.biopsycho.2018.08.001 30077768

[B27] Tik M. Hoffmann A. Sladky R. Tomova L. Hummer A. Navarro de Lara L. (2017). Towards understanding rTMS mechanism of action: stimulation of the DLPFC causes network-specific increase in functional connectivity. *Neuroimage* 162 289–296. 10.1016/j.neuroimage.2017.09.022 28912081

[B28] Wang J. X. Rogers L. M. Gross E. Z. Ryals A. J. Dokucu M. E. Brandstatt K. L. (2014). Targeted enhancement of cortical-hippocampal brain networks and associative memory. *Science* 345 1054–1057. 10.1126/science.1252900 25170153 PMC4307924

[B29] Weigand A. Horn A. Caballero R. Cooke D. Stern A. P. Taylor S. F. (2018). Prospective validation that subgenual connectivity predicts antidepressant efficacy of transcranial magnetic stimulation sites. *Biol. Psychiatry* 84 28–37. 10.1016/j.biopsych.2017.10.028 29274805 PMC6091227

[B30] Whitfield-Gabrieli S. Ghosh S. S. Nieto-Castanon A. Saygin Z. Doehrmann O. Chai X. J. (2016). Brain connectomics predict response to treatment in social anxiety disorder. *Mol. Psychiatry* 21 680–685. 10.1038/mp.2015.109 26260493

[B31] Whitfield-Gabrieli S. Nieto-Castanon A. (2012). Conn: a functional connectivity toolbox for correlated and anticorrelated brain networks. *Brain Connect.* 2 125–141. 10.1089/brain.2012.0073 22642651

[B32] Yeo B. T. Krienen F. M. Sepulcre J. Sabuncu M. R. Lashkari D. Hollinshead M. (2011). The organization of the human cerebral cortex estimated by intrinsic functional connectivity. *J. Neurophysiol.* 106 1125–1165. 10.1152/jn.00338.2011 21653723 PMC3174820

[B33] Zang Y. F. He Y. Zhu C. Z. Cao Q. J. Sui M. Q. Liang M. (2007). Altered baseline brain activity in children with ADHD revealed by resting-state functional MRI. *Brain Dev.* 29 83–91. 10.1016/j.braindev.2006.07.002 16919409

[B34] Zou Q. H. Zhu C. Z. Yang Y. Zuo X. N. Long X. Y. Cao Q. J. (2008). An improved approach to detection of amplitude of low-frequency fluctuation (ALFF) for resting-state fMRI: fractional ALFF. *J. Neurosci. Methods* 172 137–141. 10.1016/j.jneumeth.2008.04.012 18501969 PMC3902859

