# Supplementary material for: Dynamic Functional Connectivity Within the Fronto-Limbic Network Induced by Intermittent Theta-Burst Stimulation: A Pilot Study
Source: Front Neurosci. 2019 Sep 13;13:944. doi: 10.3389/fnins.2019.00944 (PMC6753168; doi:10.3389/fnins.2019.00944)
Supplement: Supplementary file 1 [file Data_Sheet_1.docx]

**Supplementary Materials**

1. **The selection of ROI**

A total of 74 ROIs was chosen from the Human Brainnetome Atlas, including:

1. 14 SFG regions (A8m_l, A8m_r, A8dl_l, A8dl_r, A9l_l, A9l_r, A6dl_l, A6dl_r, A6m_l, A6m_r, A9m_l, A9m_r, A10m_l, A10m_r);
2. 14 MidFG regions (A9_46d_l, A9_46d_r, IFJ_l, IFJ_r, A46_l, A46_r, A9_46v_l, A9_46v_r, A8vl_l, A8vl_r, A6vl_l, A6vl_r, A101_l, A101_r);
3. 12 IFG regions (A44d_l, A44d_r, IFS_l, IFS_r, A45c_l, A45c_r, A45r_l, A45r_r, A44op_l, A44op_r, A44v_l, A44v_r);
4. 12 OrG regions (A14m_l, A14m_r, A12_47o_l, A12_47o_r, A111_l, A111_r, A11m_l, A11m_r, A13_l, A13_r, A12_47l_l, A12_47l_r);
5. 14 CG regions (A23d_l, A23d_r, A24rv_l, A24rv_r, A32p_l, A32p_r, A23v_l, A23v_r, A24cd_l, A24cd_r, A23c_l, A23c_r, A32sg_l, A32sg_r);
6. 8 subcortical nuclei / amygdala and hippocampus (mAmyg_l, mAmyg_r, lAmyg_l, lAmyg_r, rHipp_l, rHipp_r, cHipp_l, cHipp_r).
7. **Temporal signal-to-noise ratio (tSNR) for bilateral frontal regions in each condition.**

We calculated tSNR over 4 SFG and MidFG ROIs (left SFG, left MidFG, right SFG and right MidFG) to exclude the susceptibility artefacts by the coil around the left DLPFC. Repeated measure ANOVAs were performed with 3 within-group factors of time (TP1, TP2 and TP3), region (SFG and MidFG) and hemisphere (left hemisphere (with coil) and right hemisphere (without coil)). The mean and standard error values in each ROI and in each condition are summarized in Supplementary Table 1. There were no significant main effect of hemisphere (F(1,9)=0.449, p=0.520) or time (F(2,18)=0.363, p=0.701). There was a significant main effect of region (F(1, 9)=21.214, p=0.001). The tSNR over the SFG were high than those over the MidFG. We also added the detailed information in the supplementary materials.

**Supplementary Table 1.** Temporal signal-to-noise ratio (tSNR) within bilateral superior frontal gyrus (SFG) and middle frontal gyrus (MidFG) for three resting-state sessions (mean±S.E.).

| **Time** | **Region** | **Left** | **Right** |
| --- | --- | --- | --- |
| Before iTBS | SFG | 169.11±13.54 | 171.39±12.33 |
|  | MidFG | 159.18±11.23 | 150.57±8.91 |
| Immediate after iTBS | SFG | 156.28±6.31 | 160.09±10.30 |
|  | MidFG | 150.59±8.14 | 139.53±6.68 |
| Fifteen minutes after iTBS | SFG | 162.26±10.17 | 167.82±13.12 |
|  | MidFG | 151.67±9.11 | 144.72±10.34 |
